# Circulatory C-type natriuretic peptide reduces mucopolysaccharidosis-associated craniofacial hypoplasia *in vivo*

**DOI:** 10.1371/journal.pone.0277140

**Published:** 2022-11-10

**Authors:** Marina Kashiwagi, Kazumasa Nakao, Shigeki Yamanaka, Ichiro Yamauchi, Takafumi Yamashita, Toshihito Fujii, Yohei Ueda, Mariko Yamamoto Kawai, Takuma Watanabe, Shizuko Fukuhara, Kazuhisa Bessho

**Affiliations:** 1 Department of Oral and Maxillofacial Surgery, Graduate School of Medicine, Kyoto University, Kyoto, Japan; 2 Department of Diabetes, Endocrinology and Nutrition, Graduate School of Medicine, Kyoto University, Kyoto, Japan; 3 Metabolism and Endocrinology Division of Internal Medicine, Kishiwada City Hospital, Osaka, Japan; 4 Department of Medical Secretarial Arts, Kansai Women’s College, Osaka, Japan; University of Cincinnati, UNITED STATES

## Abstract

Skeletal alterations in the head and neck region, such as midfacial hypoplasia, foramen magnum stenosis and spinal canal stenosis, are commonly observed in patients with mucopolysaccharidosis (MPS). However, enzyme replacement therapy (ERT), one of the major treatment approaches for MPS, shows limited efficacy for skeletal conditions. In this study, we analysed the craniofacial morphology of mice with MPS type VII, and investigated the underlying mechanisms promoting jaw deformities in these animals. Furthermore, we investigated the effects of C-type natriuretic peptide (CNP), a potent endochondral ossification promoter, on growth impairment of the craniofacial region in MPS VII mice when administered alone or in combination with ERT. MPS VII mice exhibited midfacial hypoplasia caused by impaired endochondral ossification, and histological analysis revealed increased number of swelling cells in the resting zone of the spheno-occipital synchondrosis (SOS), an important growth centre for craniomaxillofacial skeletogenesis. We crossed MPS VII mice with transgenic mice in which CNP was expressed in the liver under the control of the human serum amyloid-P component promoter, resulting in elevated levels of circulatory CNP. The maxillofacial morphological abnormalities associated with MPS VII were ameliorated by CNP expression, and further prevented by a combination of CNP and ERT. Histological analysis showed that ERT decreased the swelling cell number, and CNP treatment increased the width of the proliferative and hypertrophic zones of the SOS. Furthermore, the foramen magnum and spinal stenoses observed in MPS VII mice were significantly alleviated by CNP and ERT combination. These results demonstrate the therapeutic potential of CNP, which can be used to enhance ERT outcome for MPS VII-associated head and neck abnormalities.

## Introduction

Mucopolysaccharidosis (MPS) is an umbrella term for a group of progressive diseases caused by the impaired activity of specific lysosomal enzymes required for glycosaminoglycan degradation, and is clinically characterised by deafness, joint stiffness, obstructive airway disease, valvular disease, mental retardation and other manifestations [1, 2].

The estimated overall incidence of MPS is higher than 1:25,000 live births [3], and the life expectancy is dependent on the severity of the disease, but MPS patients may survive up to their 50s or 60s [4].

Craniofacial morphology is distinguished by a flat face, a depressed nasal bridge [5] and foramen magnum stenosis, which can lead to neurological symptoms [6, 7]. MPS is primarily treated with haematopoietic stem cell transplantation (HSCT) and enzyme replacement therapy (ERT) [8].

In HSCT, patients under the age of 24 months show significantly better development, and the donor is preferred to be a human leukocyte antigen (HLA) identical sibling [9]. GAG-mediated damage might be difficult to revert by ERT once it has occurred in the cranial bone and spine [10]. The efficacy of gene therapy combined with the ex vivo lentiviral modification of haematopoietic stem and progenitor cells in MPS I mice and humans has been reported, and with this method, it is possible to achieve higher levels of enzyme expression. However, this treatment strategy is associated with the risk of tumorigenesis as well as other conditions [11]. C-type natriuretic peptide (CNP), a member of the natriuretic peptide family, promotes bone growth and exerts its biological actions through guanylyl cyclase-B (GC-B) [12].

We previously reported that the CNP/GC-B system is a potent stimulator of endochondral bone growth and that CNP is a potent stimulator of the craniofacial region [13]. Based on these findings, we hypothesised that the CNP/GC-B system represents a novel therapeutic target for craniofacial hypoplasia in MPS VII. In this study, we analysed the head and neck morphology of MPS type VII model mice and then the effects of CNP and its co-treatment with ERT in these mice.

## Materials and methods

### Animals

Animal care and experiments were conducted in a facility of the Graduate School of Medicine, Kyoto University, and in accordance with institutional guidelines. All experimental procedures involving animals were approved by the Animal Research Committee, Kyoto University Graduate School of Medicine (permit number: Med Kyo 21268). All animals in this study were sacrificed using carbon dioxide gas. The experiments were conducted in accordance with ARRIVE guidelines. The checklist is provided in S1 File.

MPS VII model mice (Gusb^mps-2J^ mice) were purchased from Jackson Laboratory (Bar Harbour, ME, USA). We only used homozygous mice with less than 1% of the normal levels of GUSB activity [14]. The genetic background of this strain was C57BL/6.

Mice expressing CNP in the liver under the control of the human *SAP*–component promoter (*SAP-Nppc-Tg* mice) were generated on a C57BL/B6 background. Littermate of these mice had plasma CNP concentrations 84% higher than those of wild type (WT) mice as determined by radioimmunoassay measurements [15]. In a previous study, plasma CNP levels were approximately four-fold higher in 6-week-old *SAP-Nppc-Tg* mice than in WT mice [16]. Moreover, these mice, in which endochondral ossification was stimulated systemically, exhibited no hypotension or reduced heart weight as observed for mice with higher blood CNP levels [15]. Therefore, it is considered to be an appropriate in therapeutic effect and side effects. MPS VII mice with elevated circulatory CNP levels (MPS VII/SAP*-Nppc-Tg* mice) were created by crossing MPS VII and *SAP-Nppc-Tg* mice. The male and female mice show similar symptoms, only male mice were used as representative specimens in this study.

### Plasmid constructs

The pLIVE-Empty vector was purchased from Mirus Bio (Madison, WI, USA). The pLIVE-*Gusb* vectors were constructed as previously described [19].

### Hydrodynamic injection

Hydrodynamic injection enables gene transfer by the rapid injection of plasmid DNA through the tail vein of mice [17, 18]. Six-week-old mice were injected with plasmid DNA using a hydrodynamic injection-based procedure. The vectors were fixed at 100 μg for the pLIVE-*Gusb* vector, and the pLIVE-Empty vector was mixed to 125 μg as the total amount. As controls, C57BL/6 mice injected with 125 μg of pLIVE-Empty vector (WT mice), Gusb^mps-2J^/Gusb^mps-2J^ homozygous mice injected with 125 μg of pLIVE-Empty vector (MPS VII mice), Gusb^mps-2J^/Gusb^mps-2J^ homozygous mice injected with 100 μg of pLIVE-*Gusb* vector and 25 μg of pLIVE-Empty vector (MPS VII-GUSB mice), Gusb^mps-2J^/*SAP-Nppc-Tg* mice injected with 125 μg of pLIVE-Empty vector (MPS VII/SAP-CNP mice) and Gusb^mps-2J^/*SAP-Nppc-Tg* mice injected with 100 μg of pLIVE-*Gusb* vector and 25 μg of pLIVE-Empty vector (MPS VII-GUSB/SAP-CNP mice) were used for this study; the analysis was performed by dividing the animals into 5 groups.

We previously demonstrated that this injection protocol has no significant effect on measured parameters, such as nasal-anal length, nasal tail length, or weights in C57BL/6 WT and WT mice [19].

Further, we previously evaluated GAG content in male mice 4 weeks after injection to validate whether the introduction of the pLIVE*-Gusb* vector induced sufficient GUSB activity and revealed that GUSB overexpression reduced liver GAG content [19]. The mice were sacrificed and analysed at 12 weeks of age as described previously [20].

### Skull imaging

As previously reported [21], skull imaging and μCT data analysis were conducted according to linear measurements, Euclidean distance matrix analysis (EDMA), and the size of the foramen magnum was measured using the ImageJ software (National Institutes of Health).

### Growth curve analysis

Every week, from 6 to 12 weeks of age, the body weight, naso-anal length and naso-tail length were measured under isoflurane anaesthesia.

### Organ cultures

Organ cultures of 1-week-old mouse cranial bases treated with vehicle, 10^−6^ M CNP, or 1.5×10^−4^ M GUSB or both for 6 days were used to evaluate the spheno-occipital synchondrosis (SOS) as previously described [20, 22]; the GUSB concentration was determined in a previous study [23].

### Histological analysis

Histological analysis was conducted as previously described [20].

### Statistical analysis

Data are presented as the mean and standard deviations. Statistical analysis was performed using analysis of variance (ANOVA) with *P* < 0.05 considered statistically significant.

## Results

### Morphologic analyses of skulls and cervical vertebrae from WT, MPS VII, MPS VII/SAP-CNP and MPS VII-GUSB/SAP-CNP mice

**Fig 1:**
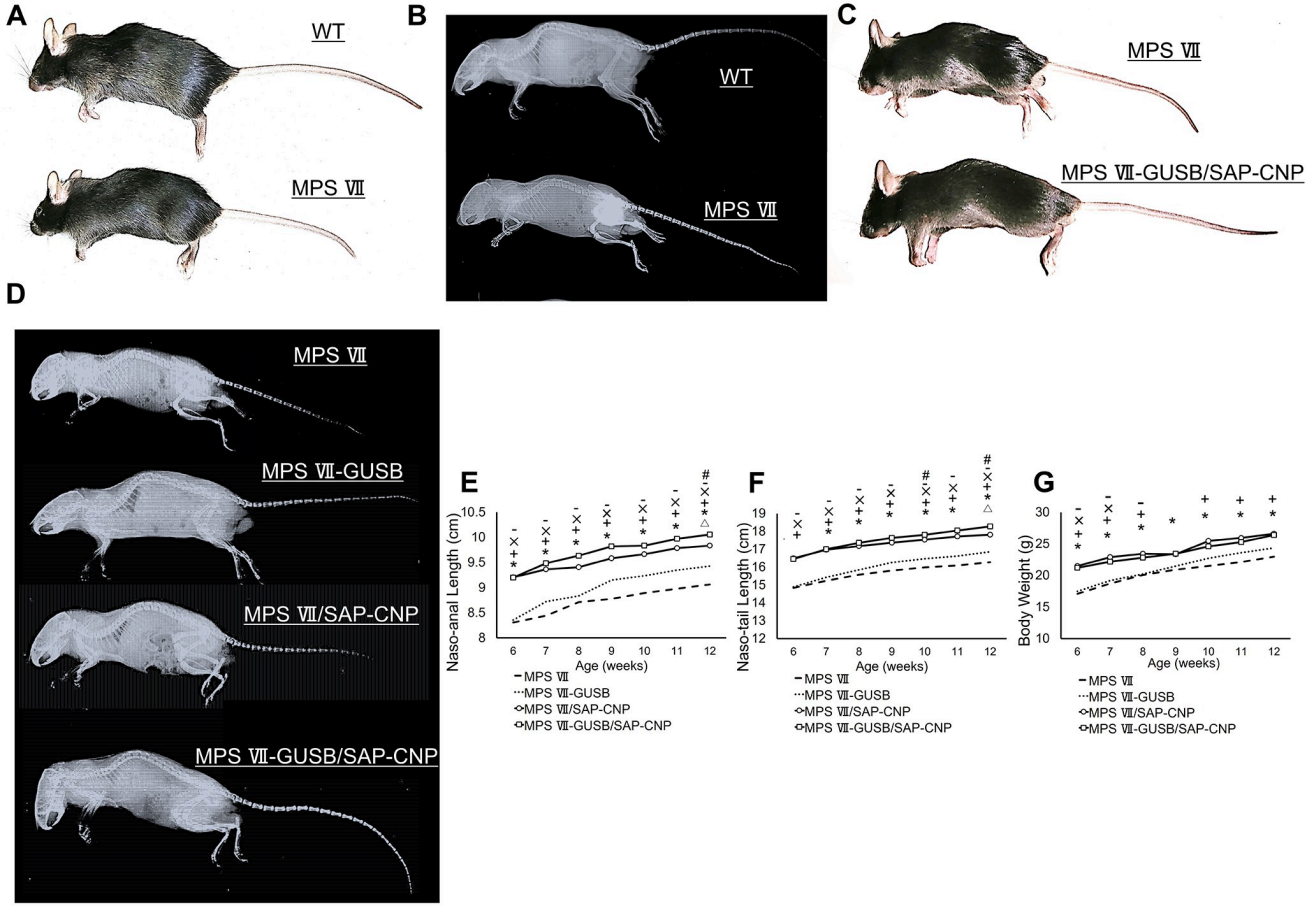
Gross morphologies and growth curves. (A) Gross morphologies of 12-week-old WT and MPS VII mice. (B) Soft X-ray pictures of 12-week-old WT and MPS VII mice. (C) Gross morphologies of 12-week-old MPS VII and MPS VII-GUSB/SAP-CNP mice. (D) Soft X-ray picture of 12-week-old MPS VII, MPS VII-GUSB, MPS VII/SAP-CNP and MPS VII-GUSB/SAP-CNP mice. Growth curves based on (E) naso-anal length, (F) naso-tail length and (G) body weight of MPS VII (dashed lines), MPS VII-GUSB (dotted lines), MPS VII/SAP-CNP (black open round lines) and MPS VII-GUSB/SAP-CNP (black open square lines) mice were generated every week from 6 to 12 weeks of age (Male, n = 7 each). *P < 0.01 for MPS VII-GUSB/SAP-CNP vs MPS VII, +P < 0.01 for MPS VII/SAP-CNP vs MPS VII, ΔP < for MPS VII-GUSB vs MPS VII, ×P < for MPS VII-GUSB/SAP-CNP vs MPS VII-GUSB, -P < for MPS VII/SAP-CNP vs MPS VII-GUSB, #P < for MPS VII-GUSB/SAP-CNP vs MPS VII/SAP-CNP.

As previously described [24], MPS VII mice exhibited dwarfism and short limb bones, with gross morphologies and soft X-ray images (Fig 1A and 1B). MPS VII-GUSB/SAP-CNP mice had the largest body length among the various treatment groups (Fig 1C and 1D). As demonstrated by the growth curves, naso-anal and naso-tail lengths were significantly decreased in MPS VII mice in all treatment groups, slightly increased in MPS VII-GUSB mice and highly increased in MPS VIIsapCNP-Empty mice, and the lengths in MPS VII-GUSB/SAP-CNP mice were significantly longer than those in mice in all other injection groups at 12 weeks of age (Fig 1E and 1F). The body weights of MPS VII/SAP-CNP and MPS VII-GUSB/SAP-CNP mice were significantly higher than those of MPS VII mice at 12 weeks of age (Fig 1G).

**Fig 2:**
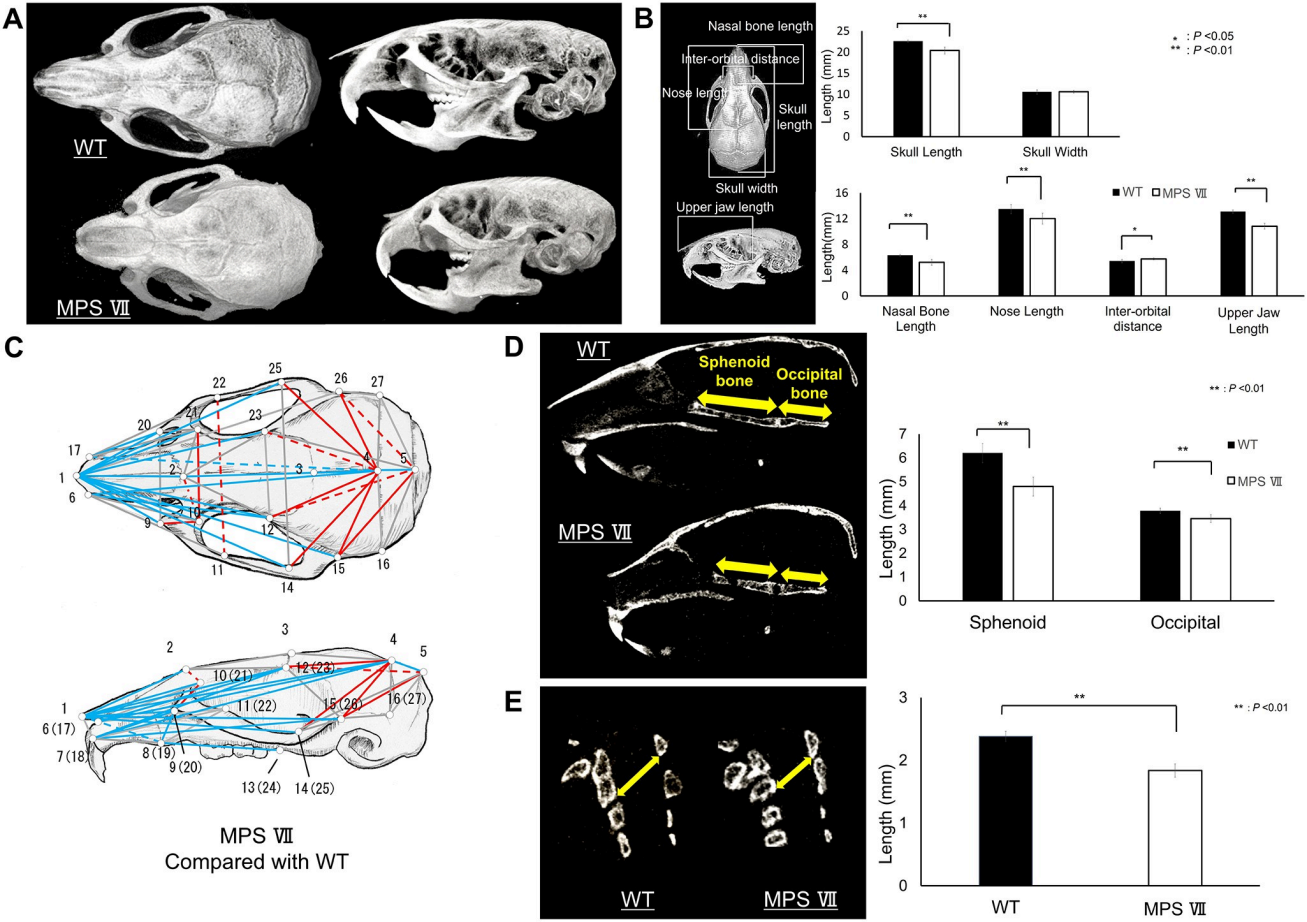
Head and neck morphology of WT and MPS VII mice. (A) Three-dimensional reconstructed images of the skulls of 12-week-old WT and MPS VII mice. (B) Linear measurements for the analysis of normal mouse skulls (left pictures). Linear measurements of 12-week-old WT and MPS VII (n = 7) mice (P < 0.05 or 0.01) (right graph). (C) Landmarks used for EDMA. Schematic images of the mouse cranium (upper, superior view; middle, lateral view). Significantly smaller or larger values (n = 7, P < 0.05 or 0.01) for MPS VII mice compared with those for WT mice are denoted by different lines. Blue lines show significant hypoplasia and red lines show significant hyperplasia compared with WT mice (solid lines, P < 0.01; dotted lines, P < 0.05). (D) Sagittal sections from micro–computed tomography (μCT) images of 12-week-old WT and MPS VII mice and sagittal lengths of occipital and sphenoid bones of 12-week-old WT and MPS VII mice (n = 7 each, P < 0.01). (E) Cervical vertebrae on μCT images and lengths from the posterior and inferior aspects of the body of cervical vertebra 2 to the inferior aspect of the arch of cervical vertebra 1 of 12-week-old WT and MPS VII mice (n = 7 each, P < 0.01).

Morphometric analysis of the crania was performed using μCT images (Fig 2A). Linear measurements revealed that the skull, nasal bone, nose and upper jaw lengths in MPS VII mice were significantly shorter than those in WT mice (Fig 2B). Conversely, inner canthal distances were significantly larger in the MPS VII crania than in the WT crania (Fig 2B). Furthermore, EDMA revealed that the nasal, premaxilla, maxilla and frontal bones were markedly affected sagittally, resulting in hypoplasia in the MPS VII crania (Fig 2C). In contrast, the cranial width was larger in the MPS VII crania than in the WT crania (Fig 2C). Measurements of EDMA are in the S2 File. μCT imaging revealed that both the occipital and sphenoid bones that make up the skull base were significantly shorter in MPS VII mice than in WT mice (Fig 2D). In spinal canal measurements, μCT imaging also revealed that lengths from the posterior and inferior aspects of the body of cervical vertebra 2 to the inferior aspect of the arch of cervical vertebra 1 in MPS VII mice were significantly shorter than those in WT mice (Fig 2E).

**Fig 3:**
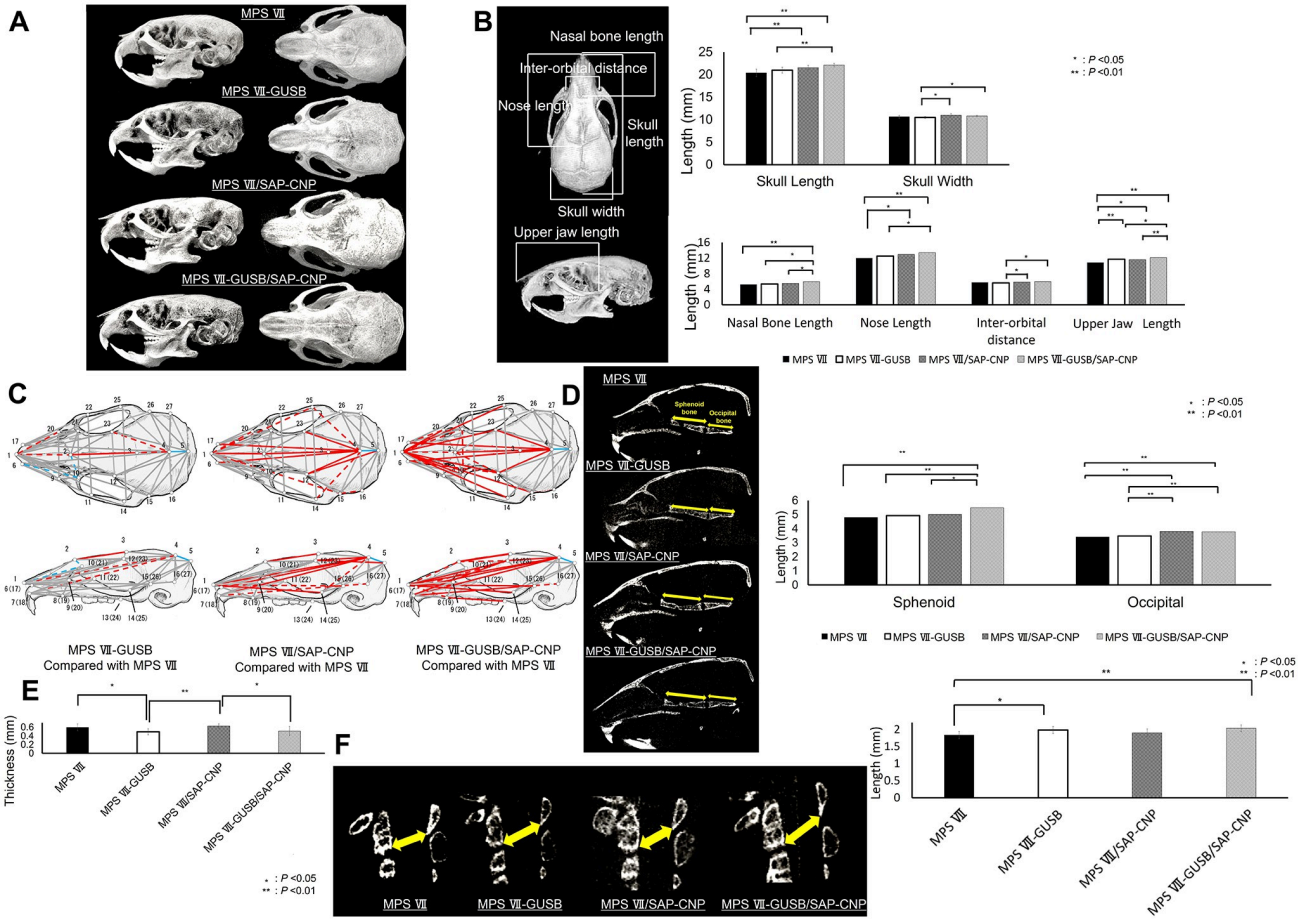
Head and neck morphology of MPS VII, MPS VII-GUSB, MPS VII/SAP-CNP and MPS VII-GUSB/SAP-CNP mice. (A) Three-dimensional reconstructed images of the skulls of 12-week-old MPS VII, MPS VII-GUSB, MPS VII/SAP-CNP and MPS VII-GUSB/SAP-CNP mice. (B) Linear measurements for analysis of normal mouse skulls (left). Linear measurements from 12-week-old MPS VII, MPS VII-GUSB, MPS VII/SAP-CNP and MPS VII-GUSB/SAP-CNP mice (n = 7 each, P < 0.05 or 0.01) (right). (C) Landmarks used for Euclidean distance matrix analysis (EDMA). Schematic images of the mouse cranium. MPS VII-GUSB mice compared with MPS VII mice, MPS VII/SAP-CNP mice compared with MPS VII mice, MPS VII-GUSB/SAP-CNP mice compared with MPS VII mice. Significantly smaller or larger values (n = 7, P < 0.05 or 0.01) compared with those for MPS VII mice are denoted by different lines. Blue lines show significant hypoplasia and red lines show significant hyperplasia compared to those in the MPS VII mice (solid lines, P < 0.01; dotted lines, P < 0.05). (D) Sagittal sections from μCT images and sagittal lengths of occipital and sphenoid bones from 12-week-old MPS VII, MPS VII-GUSB, MPS VII/SAP-CNP and MPS VII-GUSB/SAP-CNP mice (n = 7 each, P < 0.05 or 0.01). (E) Lengths of the SOS from μCT images of 12-week-old MPS VII, MPS VII-GUSB, MPS VII/SAP-CNP and MPS VII-GUSB/SAP-CNP mice (n = 7 each, P < 0.05 or 0.01). (F) Cervical vertebrae on μCT images and lengths from the posterior and inferior aspects of the body of cervical vertebra 2 to the inferior aspect of the arch of cervical vertebra 1 of 12-week-old MPS VII, MPS VII-GUSB, MPS VII/SAP-CNP and MPS VII-GUSB/SAP-CNP mice (n = 7 each, P < 0.05 or 0.01).

μCT images were used for skull morphometric analyses to evaluate the effects of GUSB, CNP, or both on MPS VII (Fig 3A). Linear measurements showed significantly longer skull, nasal bone, nose and upper jaw lengths in MPS VII-GUSB/SAP-CNP mice. The upper jaw length was also significantly increased in MPS VII-GUSB mice, while the skull, nose and upper jaw lengths were significantly increased in MPS VII/SAP-CNP mice (Fig 3B). EDMA revealed that the hypoplasia observed in the MPS VII skulls was slightly alleviated in the MPS VII-GUSB skulls, markedly alleviated in the MPS VII/SAP-CNP skulls and most notably alleviated in the MPS VII-GUSB/SAP-CNP skulls (Fig 3C). Furthermore, the sagittal length of sphenoid bones was significantly increased in MPS VII-GUSB/SAP-CNP mice, and the lengths of occipital bones were also increased in MPS VII/SAP-CNP and MPS VII-GUSB/SAP-CNP mice (Fig 3D). On μCT, sagittal thickness measurements of the SOS showed significant thinning in the presence of GUSB (Fig 3E). Spinal stenosis was significantly alleviated in MPS VII-GUSB mice and most alleviated in MPS VII-GUSB/SAP-CNP mice (Fig 3F).

### Histological analyses of the skull base in WT and MPS VII mice

**Fig 4:**
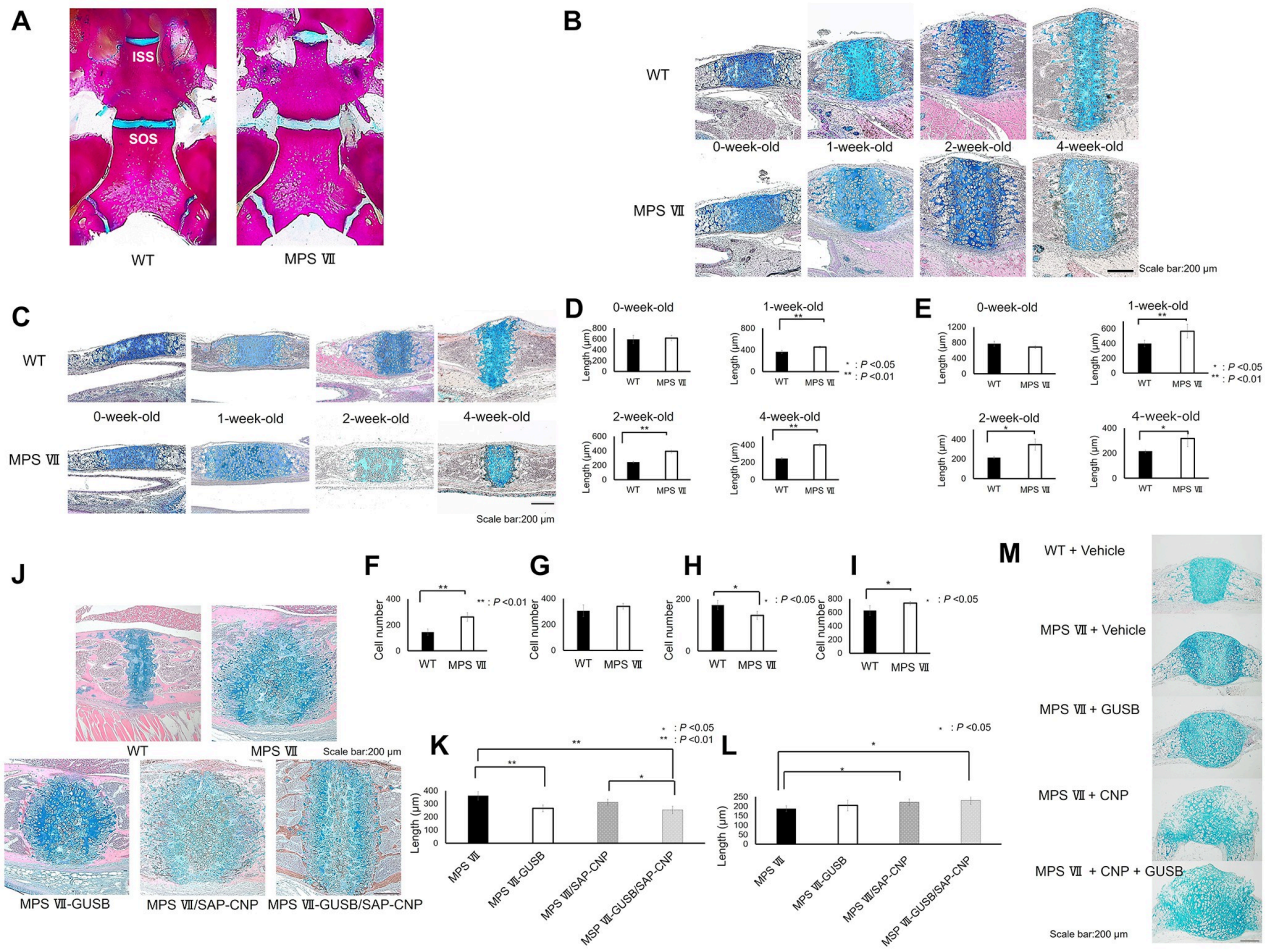
Histological analyses of SOS and ISS. (A) Alizarin red and Alcian blue staining of 2-week-old skull bases in WT and MPS VII mice. (B, C) Alcian blue/haematoxylin and eosin (HE) staining in sagittal sections of the SOS (B) and (C) ISS of 0, 1, 2, 4-week-old WT and MPS VII mice (n = 4 each, *P* < 0.05 or 0.01). (D, E) Histological lengths of SOS (D) and ISS (E). (F-I) Cell number in the resting zone (F), proliferative zone (G) and hypertrophic zone (H) and overall SOS (I) in 4-week-old WT and MPS VII mice (n = 4 each, *P* < 0.05 or 0.01). (J) Alcian blue/HE staining of 12-week-old skull bases in WT, MPS VII, MPS VII-GUSB, MPS VII/SAP-CNP and MPS VII-GUSB/SAP-CNP mice. (K) Width of the resting zone of the SOS from 12-week-old MPS VII, MPS VII-GUSB, MPS VII/SAP-CNP and MPS VII-GUSB/SAP-CNP mice (n = 4 each, *P* < 0.05 or 0.01). (L) Width of proliferative and hypertrophic zones of the SOS from 12-week-old MPS VII, MPS VII-GUSB, MPS VII/SAP-CNP and MPS VII-GUSB/SAP-CNP mice (n = 4 each, *P* < 0.05 or 0.01). (M) Histological examination of the skull base after a 6-day organ culture and staining with Alcian blue/HE in skull base explants from 1-week-old WT and MPS VII mice (n = 2 each).

To understand the mechanism underlying hypoplasia in MPS VII mice, we analysed the SOS and inter-sphenoid synchondrosis (ISS) of the cranial base, which are growth centres of craniofacial skeletogenesis and contribute to the longitudinal length of the cranial base [25, 26]. WT and MPS VII mouse skull base preparations of 2-week-old were stained with Alizarin red and Alcian blue in the horizontal positions and sagittal sections of 0, 1, 2 and 4-week-old mice were stained with Alcian blue/HE. Alizarin red and Alcian blue staining revealed that the skull bases of 2-week-old MPS VII mice had thicker synchondroses than those of WT mice (Fig 4A). ISS and SOS with swelling cells in the resting zone were observed in after 1-week-old MPS VII mice (Fig 4B and 4C). Alcian blue/HE-stained sagittal sections from 0-week-old mice showed no significant difference in the thickness of SOS and ISS between WT and MPS VII mice, but in 1-, 2- and 4-week-old mice, they were significantly thicker in MPS VII mice than in WT mice (Fig 4D and 4E). When MPS VII mice were compared with WT mice, the number of cells in the resting zone was significantly increased, the number of cells in the hypertrophic zone was significantly decreased and the overall cell number of the SOS was significantly increased in the MPS VII mice (Fig 4F–4I).

### Histological analyses of the skull base in WT, MPS VII, MPS VII-GUSB, MPS VII/SAP-CNP and MPS VII-GUSB/SAP-CNP mice

Despite hypogrowth of the midface in the sagittal direction in 12-week-old mouse skull bases, the SOS was thicker in MPS VII mice than in WT mice (Fig 4J). The GUSB treatment slightly alleviated the swelling cells, and the resting zone became thinner (Fig 4j and 4K).

In MPS VII/SAP-CNP mice, the proliferative and hypertrophic zones became significantly thicker, but the resting zones did not become significantly thinner than those in MPS VII mice (Fig 4J–4L). When compared with those in MPS VII mice, the resting zones in MPS VII-GUSB/SAP-CNP mice became significantly thinner, while the proliferative and hypertrophic zones became significantly thicker (Fig 4J–4L).

### Organ culture experiments of the skull base from WT and MPS VII mice

To evaluate the combined effects of CNP and GUSB on SOS growth, we performed organ culture experiments using skull base explants from 1-week-old MPS VII mice and WT mice because the resting zone became significantly thicker and GAGs recognised the accumulation. Compared to WT explants, the vehicle-treated MPS VII explants showed swelling cells in the resting zone and increased SOS thickness after a 6-day culture but decreased cell numbers in the proliferative and hypertrophic zones. In addition, compared to vehicle-treated MPS VII explants, GUSB-treated MPS VII explants showed an increased number of cells in the proliferative and hypertrophic zones. CNP-treated MPS VII explants showed enlarged chondrocytes in the proliferative and hypertrophic zones, and CNP- and GUSB-treated MPS VII explants showed increased numbers of enlarged cells and increased cell numbers in the proliferative and hypertrophic zones, respectively (Fig 4M).

### Morphologic analyses of the foramen magnum of WT, MPS VII, MPS VII-GUSB, MPS VII/SAP-CNP and MPS VII-GUSB/SAP-CNP mice

**Fig 5:**
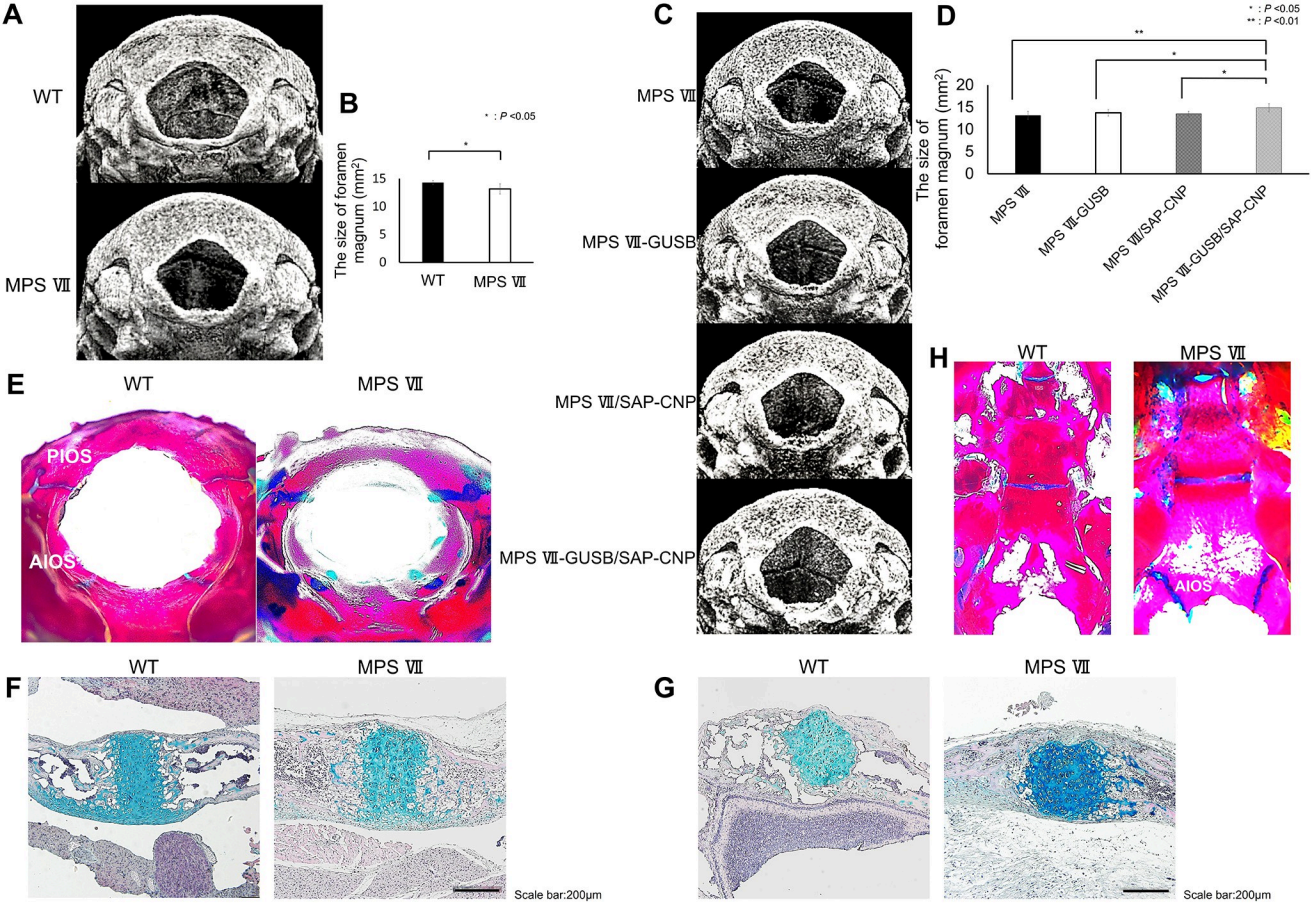
Effect of CNP and GUSB on foramen magnum stenosis. (A) Three-dimensional reconstructed images of the foramen magnum of 12-week-old WT and MPS VII mice. (B) Sizes of the foramen magnum of 12-week-old WT and MPS VII mice. (n = 7 each, *P* < 0.05). (C) Three-dimensional reconstructed images of the foramen magnum of 12-week-old MPS VII, MPS VII-GUSB, MPS VII/SAP-CNP and MPS VII-GUSB/SAP-CNP mice. (D) Sizes of the foramen magnum of 12-week-old MPS VII, MPS VII-GUSB, MPS VII/SAP-CNP and MPS VII-GUSB/SAP-CNP mice (n = 7 each, *P* < 0.05 or 0.01). (E) Alizarin red and Alcian blue staining of the foramen magnum in 2-week-old WT and MPS VII mice. (F, G) Alcian blue/HE staining of the AIOS (F) and PIOS (G) of 2-week-old WT and MPS VII mice. (H) Alizarin red and Alcian blue staining of 12-week-old skull bases of WT and MPS VII mice.

To investigate whether CNP and GUSB are effective treatments for foramen magnum stenosis observed in MPS VII, we compared this region in WT and MPS VII mouse skulls (Fig 5A). At 12 weeks of age, the foramen magnum was significantly smaller (–7.827%) in MPS VII skulls than in WT skulls (Fig 5B). The MPS VII-GUSB/SAP-CNP mice had the largest foramen magnum (Fig 5C and 5D), while MPS VII-GUSB or MPS VII/SAP-CNP mice did not differ significantly from MPS VII mice in foramen magnum.

### Histological analysis of the foramen magnum in WT and MPS VII mice

The foramen magnum is formed by the exoccipital, supraoccipital and basioccipital bones and grows through endochondral ossification [27]. The anterior intraoccipital synchondrosis (AIOS) exists between the exoccipital and basioccipital bones, and the posterior intraoccipital synchondrosis (PIOS) exists between the exoccipital and supraoccipital bones.

At 2 weeks of age, Alizarin red and Alcian blue staining revealed thicker AIOS and PIOS in MPS VII mice than in WT mice (Fig 5E). In Alcian blue/HE staining, MPS VII mice had thicker AIOS and PIOS, and their chondrocyte columns were not in order when compared to those in WT mice (Fig 5F and 5G). The skull base in 12-week-old mice showed that AIOS remained in MPS VII mice, but was closed in WT mice (Fig 5H).

## Discussion

In this study, we analysed the morphology of the head and neck region in MPS VII mice and found that MPS VII mice exhibit a characteristic craniofacial morphology, including midface hypoplasia, foramen magnum stenosis and spinal canal stenosis, similar to that observed in patients with MPS.

Various mechanisms of skeletal growth inhibition in MPS have been reported. In patients with MPS, endochondral ossification at the growth plates fails, resulting in reduced chondrocyte proliferation and delayed differentiation from proliferation to hypertrophy in animal models [28].

Previous studies reported that the growth plates of MPS VII model mice with short stature were thicker than those of normal mice [29]. The increase in growth plate thickness was caused by an increase in the number of cells in the resting zone as well as an increase in chondrocyte size and spacing due to GAG storage.

As previously reported [19], the resting zone in the tibial growth plates of MPS model mice contained swelling cells. Swelling cells in the resting zone may have had impaired function, and the number of chondrocytes from the resting zone may have been reduced, resulting in growth inhibition.

Furthermore, a disorganised structure in the hypertrophic zone was observed, in addition to an abnormal arrangement of the primary trabecular bone and an increased presence of cartilage within the woven bone, suggesting difficulties in cartilage resorption during endochondral ossification in MPS model mice [30].

On the other hand, increased chondrocyte apoptosis in MPS was revealed, and chondrocyte culture experiments of MPS may compensate for the increased chondrocyte apoptosis and increased cell proliferation. The reason for the significant bone abnormalities observed despite this compensation mechanism is that immunostaining revealed that the level of osteonectin, a marker of mature chondrocytes, is significantly reduced in the tibial growth plate of MPS animals, suggesting a defect in bone production due to a reduction in hypertrophic chondrocytes available for mineralisation into bone [31].

This study revealed that the mechanisms of inhibition of long bone growth and midface hypoplasia are similar. Histological analysis revealed that MPS VII model mice had thicker SOS and ISS, which are important for the midfacial sagittal growth given that the columns of chondrocytes are oriented roughly parallel to the longitudinal axis of the basicranium. After one week, swelling was observed in the resting zone, and the number of cells in the hypertrophic zone was lower than that in WT mice. This suggests that functional resting zone cells are not being delivered to other zones in the SOS and ISS as well as long bones.

In this rescue experiment involving crossbreeding of MPS VII and *SAP-Nppc-Tg* mice and the vector administration, MPS VII-GUSB mice showed slight alleviation, MPS VII/SAP-CNP mice showed moderate alleviation and MPS VII-GUSB/SAP-CNP mice showed the most remarkable alleviation of maxillofacial morphological abnormalities. This suggests that the co-treatment of CNP and ERT was the most effective treatment.

Histology and organ cultures suggest that GUSB decreased the swelling of the resting zone and increased the number of cells in proliferative and hypertrophic zones but did not increase the width of the proliferative and hypertrophic zones sufficiently. CNP increased the number of enlarged cells in proliferative and hypertrophic zones, as did GUSB and CNP treatment. In addition, we previously revealed that GUSB therapy significantly increased *Npr2* mRNA levels [19]; this study also showed that the co-treatment of ERT and CNP may have synergistic effects.

We previously reported that MPS VII mice with elevated blood levels of CNP from 6 weeks of age failed to show improvements in short stature [19]; however, in the present study, elevated blood levels of CNP from birth predominantly alleviated short stature in MPS VII mice. This difference is due to CNP expression timing, and it is considered that CNP acts effectively at birth when GAG accumulation is low, and CNP may become resistant to GAG accumulation. This suggests that early CNP treatment could be used to treat skeletal abnormalities associated with MPS VII.

A previous study reported that in MPS type II patients who were treated with ERT at 3 months of age, the only physical sign of the disease was a mild deformity of one vertebra, and they did not exhibit short stature or coarse facial features after 3 years of treatment [32]; however, in this study, MPS VII-GUSB mice did not show sufficient improvement in short stature or midface hypoplasia. This may be due to the late timing of ERT and the fact that MPS VII mice exhibit more severe skeletal abnormalities than mice with other MPS types [33].

Spinal canal stenosis and foramen magnum stenosis are characteristic findings of patients with MPS [34, 35], and MPS VII model mice showed spinal canal and foramen magnum stenosis in this study. In terms of foramen magnum stenosis, Alizarin red and Alcian blue staining of the skull base in 12-week-old mice revealed that the AIOS was still present in MPS VII mice but was closed in WT mice; this delayed ossification may have contributed to foramen magnum stenosis. Furthermore, combined treatment with GUSB and CNP alleviated the narrowed spinal canal and foramen magnum stenosis in MPS VII mice.

In conclusion, CNP may be effective, while a combination of GUSB and CNP may be more effective, in treating impaired skeletogenesis in patients with MPS, including not only short stature but also craniofacial hypoplasia, narrowing of the foramen magnum and spinal canal stenosis.

## Supporting information

S1 File The ARRIVE guidelines checklist. (DOCX)

S2 File The measurements of EDMA. (XLSX)
