# Supplementary material for: Circulatory C-type natriuretic peptide reduces mucopolysaccharidosis-associated craniofacial hypoplasia in vivo
Source: PLoS One. 2022 Nov 10;17(11):e0277140. doi: 10.1371/journal.pone.0277140 (PMC9648782; doi:10.1371/journal.pone.0277140)

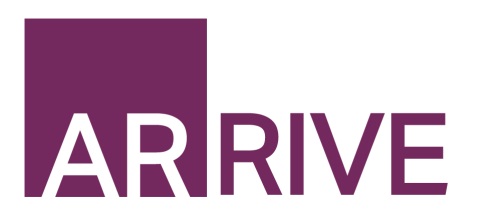


The ARRIVE Guidelines Checklist

Animal Research: Reporting In Vivo Experiments

Marina Kashiwagi ^1^, Kazumasa Nakao^1＊^, Shigeki Yamanaka^1^, Ichiro Yamauchi^2^, Takafumi Yamashita^3^, Toshihito Fujii^2^, Yohei Ueda^2^, Mariko Yamamoto Kawai^1,4^, Takuma Watanabe^1^, Shizuko Fukuhara^1^, Kazuhisa Bessho^1^

*^1^Department of Oral and Maxillofacial Surgery, Graduate School of Medicine, Kyoto University, Kyoto, Japan, ^2^Department of Diabetes, Endocrinology and Nutrition, Graduate School of Medicine, Kyoto University, Kyoto, Japan, ^3^Metabolism and Endocrinology Division of Internal Medicine, Kishiwada City Hospital, Osaka, Japan, ^4^Department of Medical Secretarial Arts, Kansai Women’s College, Osaka, Japan.*

|  | | ITEM | RECOMMENDATION | Section/ Paragraph |
| --- | --- | --- | --- | --- |
| 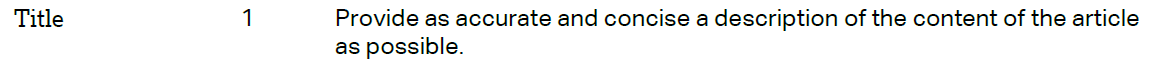 | | | Title |  |
| 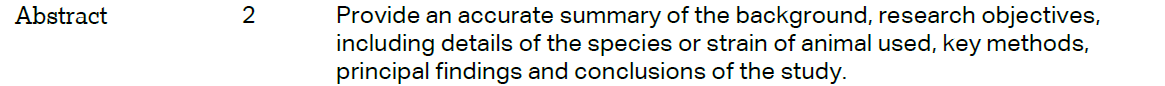 | | | Abstract,  Materials and Methods |  |
| INTRODUCTION | | |  |  |
| 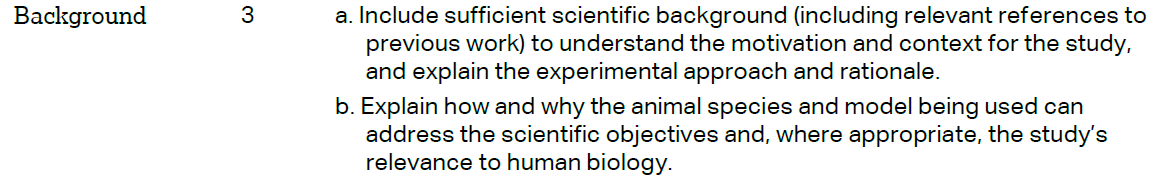 | | | Introduction,  Materials and Methods |  |
| 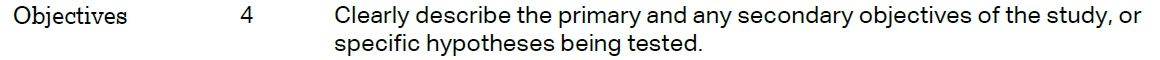 | | | Introduction |  |
| METHODS | | |  |  |
| 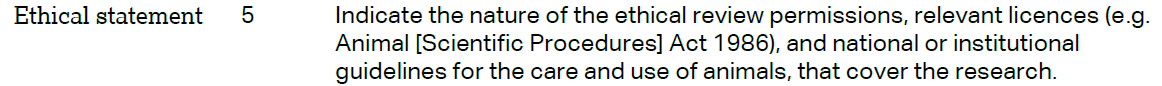 | | | Paragraph 6 |  |
| 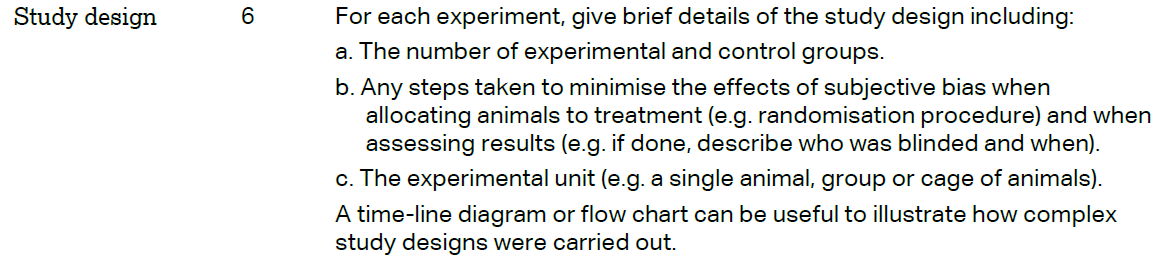 | | | Figure Captions,  Paragraphs 10 |  |
| 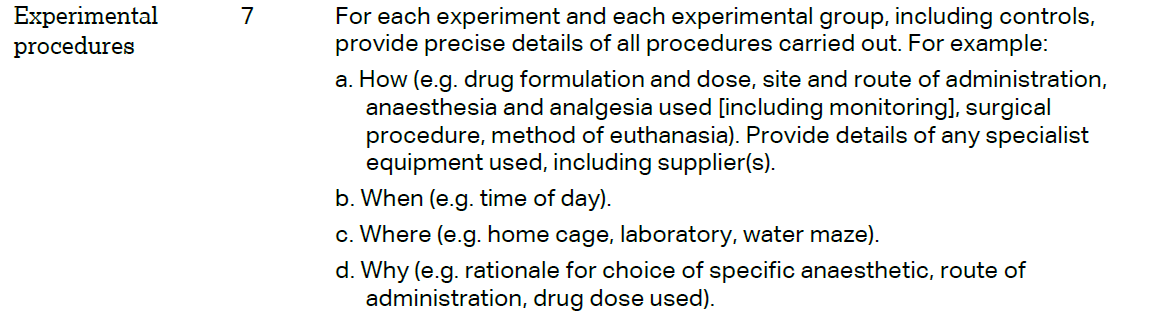 | | | Paragraphs 12 ,14 |  |
| 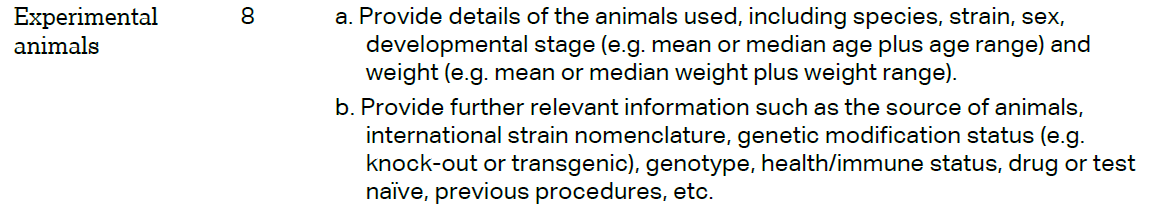 | | | Figure Captions,  Paragraphs 7,8,10 |  |

The ARRIVE guidelines. Originally published in *PLoS Biology*, June 2010^1^

| 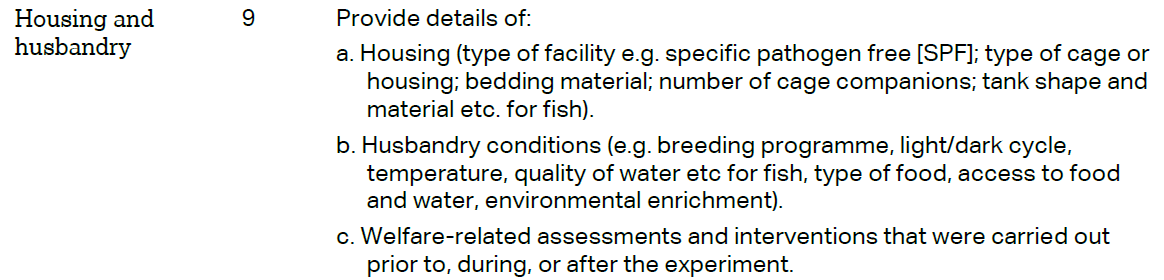 | Materials and Methods | |
| --- | --- | --- |
| 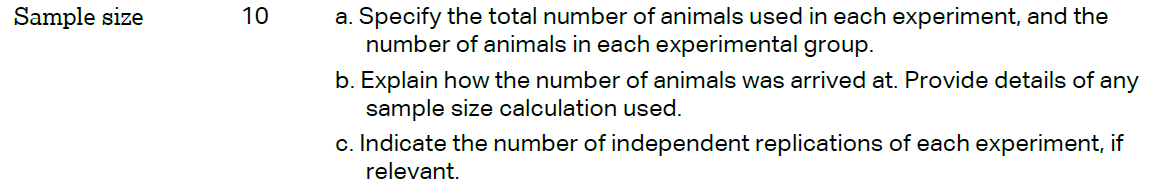 | Figure Captions,  Paragraph 10 | |
| 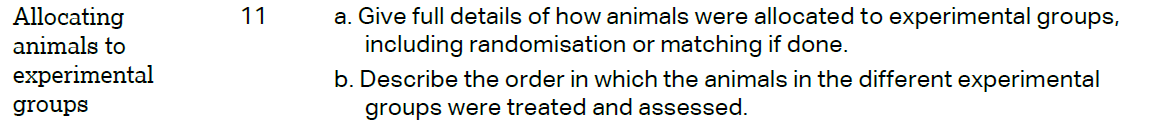 | Materials and Methods,  Figure Captions, | |
| 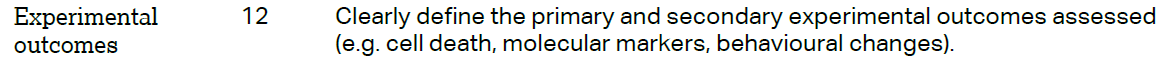 | Results | |
| 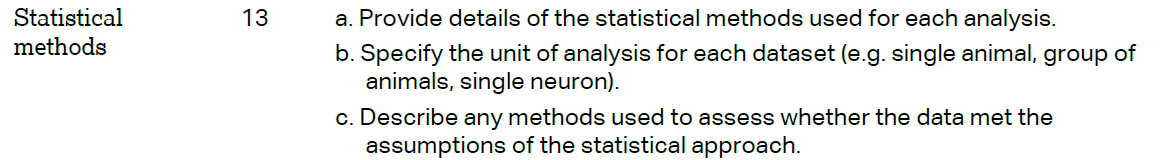 | Paragraph 17 | |
| RESULTS |  | |
| 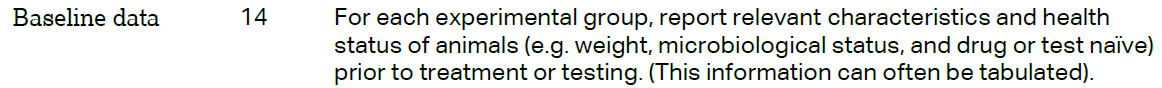 | Paragraph 6 | |
| 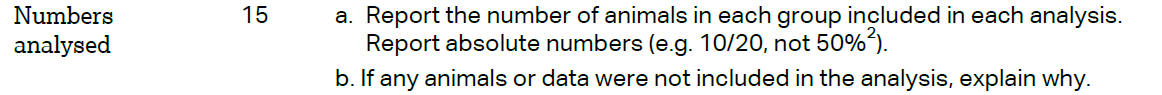 | Figure Captions | |
| 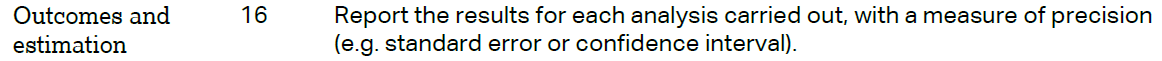 | Paragraph 17, Figures | |
| 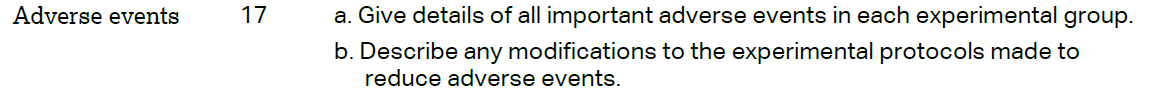 | Paragraph 12 | |
| DISCUSSION |  | |
| 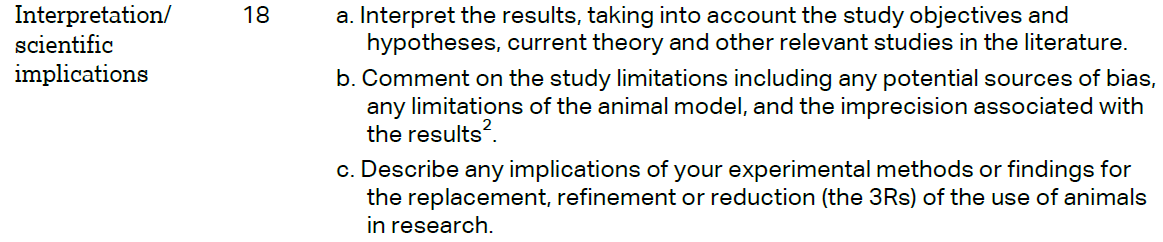 | Paragraph 10, Discussion | |
| 1-3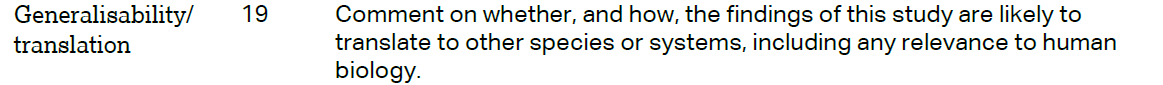 | Paragraphs 4-5 | |
| 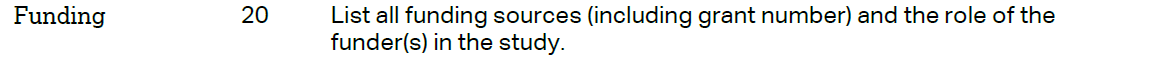 | | Funding Information |


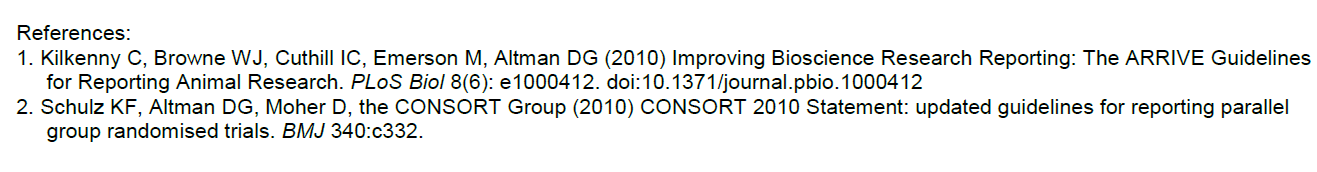

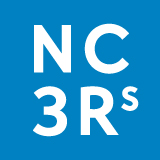

Supplement: S1 File — (DOCX) [file pone.0277140.s001.docx]
